# Target Engagement in a Head-to-head Clinical Trial of Dysphoric versus Anxiosomatic TMS Targets

**DOI:** 10.21203/rs.3.rs-7255101/v1

**Published:** 2025-08-27

**Authors:** Samantha Baldi, Christopher Lin, Jing Li, Summer Frandsen, Emma Jones, Stephan Palm, Abigail Greene, Joseph Taylor, Michael Fox, Shan Siddiqi

**Affiliations:** Brigham and Women’s Hospital, Harvard Medical School; Brigham and Women’s Hospital; Brigham and Women’s Hospital; Brigham and Women’s Hospital; Brigham and Women’s Hospital; Brigham and Women’s Hospital; Harvard University; Mass General Brigham, Harvard Medical School; Brigham and Women’s Hospital, Harvard Medical School; Harvard Medical School, Brigham & Women’s Hospital

## Abstract

Functional connectivity (FC) is increasingly used to measure target engagement following transcranial magnetic stimulation (TMS), but whether FC changes are specific to the TMS target remains unclear. We examined FC before and after TMS in patients with depression and anxiety (N=29) enrolled in a randomized trial targeting a dysphoric vs. anxiosomatic circuit. We tested whether: 1) TMS selectively modulated FC within the targeted circuit; 2) baseline TMS site FC to the targeted circuit predicted FC changes; 3) either metric correlated with symptom improvement. We found that: 1) FC decreased in both circuits, but effects were not clearly target-specific; 2) FC to the targeted circuit correlated with FC changes; 3) FC to the targeted circuit, not FC changes, correlated with symptom improvement. Results were driven by the anxiosomatic target group, likely due to heterogeneity in dysphoric circuit properties. These results have implications for using FC to target TMS and measure engagement.

## Introduction

Transcranial magnetic stimulation (TMS) is delivered clinically to cortical sites based on neurobiological models and imaging evidence that link those sites to the disorder under treatment.^1,2^ Variability in treatment response is often attributed to individual differences in targeting – i.e., whether the stimulation pulse was delivered over the intended cortical area^2^ – and target engagement – i.e., whether stimulation actually perturbs the region or circuit thought to mediate symptom change.^3^

In motor cortex studies, target engagement is straightforward: a TMS pulse elicits a motor response that provides immediate feedback on whether the targeted region was engaged. Outside the motor cortex, concurrent (EEG)-TMS-fMRI approaches have enabled real-time measurement of stimulation-induced neural and behavioral/cognitive changes. These studies have been instrumental in demonstrating that TMS can produce remote engagement of distributed networks^4^, and that these effects are strongly state-dependent^3^. However, due to their technical complexity, such methods are typically limited to single-session experiments in highly controlled settings. In psychiatric applications – where TMS is delivered repeatedly over weeks – there remains no consensus approach to evaluating target engagement.^5^ Researchers often rely on indirect measures, such as pre-to-post treatment changes in brain signal within a region or circuit, as measured by neuroimaging or electrophysiology. However, while this approach may demonstrate engagement, it does not necessarily establish target-dependence: neural changes can arise from non-specific treatment effects, independently of the specific stimulation target. This distinction is particularly important in mechanistic clinical trials, where verifying target-dependent engagement can help determine if symptom change – or lack thereof – reflects the hypothesized mechanism rather than some alternative factor.

Because TMS effects extend to brain circuits functionally connected to the cortical target site^6^, resting-state functional connectivity (FC) can both define circuit-based targets and verify whether these targets were engaged with treatment. By leveraging incidental variation in TMS target location, we retrospectively showed that “dysphoric” symptoms of depression – e.g., sadness, anhedonia, suicidality – improved more when stimulating one brain circuit functionally connected to left dorsolateral prefrontal (dlPFC) targets, while “anxiosomatic” symptoms – e.g., worry, irritability, insomnia – improved more when stimulating a distinct circuit associated with more dorsomedial (dmPFC) targets.^7^ These findings led to two symptom-specific circuit targets: a “dysphoric” circuit target centered in the left dlPFC, and an “anxiosomatic” circuit target centered in the dmPFC.^7^ In a subsequent head-to-head clinical trial in patients with depression and anxiety, patients randomized to either the dysphoric or the anxiosomatic target showed differential improvement in depression versus anxiety, respectively, supporting the rationale for symptom-specific TMS targeting.^8^

This two-arm trial design, targeting two distinct brain circuits, provides the opportunity to relate neurophysiological changes to a specific stimulation target, and to determine whether clinical effects arose from target-dependent engagement of the underlying circuits. We first conducted a preliminary analysis in an independent cohort receiving clinical TMS, where incidental variability in stimulation site location allowed us to examine how FC between the TMS site and an underlying circuit relate to FC changes. Findings from this analysis then informed pre-specified hypotheses in the head-to-head trial. Using pre- and post-treatment resting-state FC data, we directly examined whether: (1) TMS at the dysphoric or anxiosomatic target selectively modulated FC within the corresponding circuit; (2) baseline FC between the TMS site and the intended circuit predicted the magnitude of circuit engagement; and (3) metrics of target engagement and baseline TMS site FC correlated with clinical outcomes.

## Results

### Preliminary analyses on circuit-specific FC changes

To assess whether FC changes could serve as a biomarker of target-dependent engagement, we conducted a preliminary analysis in 18 patients who underwent 28-minute resting-state fMRI scans before and after a course of clinical TMS, as part of a larger study on incidental variability in clinical TMS sites.^9^ TMS was targeted on the scalp according to the “5.5cm” rule, resulting in incidental variance in TMS site location across patients. The location of individual TMS sites was recorded using neuronavigation, as previously described.^9^ We computed average FC within each circuit following established methods,^10^ and tested whether baseline FC between the TMS site and each circuit correlated with the magnitude of FC change within that circuit.

Individualized TMS site FC to the anxiosomatic circuit was associated with FC decrease within this circuit (*r*=−0.43, *p*=0.07; *r*=−0.53, *p*=0.03 after controlling for baseline FC within the anxiosomatic circuit). This association was not found for the dysphoric circuit (*r*=−0.09, *p*=0.70; *r*=−0.10, *p*=0.69 after controlling for baseline FC within the dysphoric circuit) (**Figure S1**). Of note, this analysis is limited by the fact that 16 of the 18 patients’ TMS sites showed stronger connectivity to the anxiosomatic circuit, so there is limited potential to explain variance in the dysphoric circuit.

These preliminary results align with prior studies showing that TMS tends to reduce resting-state FC within targeted circuits,^11^ and support the use of FC decreases as a biomarker of target-dependent engagement. This rationale informed the pre-specified hypotheses in the imaging component of the head-to-head clinical trial.

### FC within the anxiosomatic circuit decreases more after TMS at the anxiosomatic target

Forty patients meeting FDA criteria for TMS for depression, and with comorbid anxiety symptoms, were randomized to TMS at either the dysphoric (MNI:[−32,44,34]) or the anxiosomatic (MNI:[0,48,46]) target, receiving the 2008 FDA-cleared treatment protocol.^8^ Four participants withdrew before completing any follow-up clinical assessment. Seven participants completed some treatments and at least one follow-up assessment, but withdrew before the final imaging visit. N=29 patients were included in the current analysis who completed a pre- and post-TMS imaging visit where structural and rs-fMRI data was collected. Thirteen participants received TMS at the dysphoric target, sixteen at the anxiosomatic target.

We tested for target-dependent FC changes, and hypothesized that FC within the anxiosomatic circuit would decrease more following TMS at the anxiosomatic target, while FC within the dysphoric circuit would decrease more following TMS at the dysphoric target. We computed the change in average FC within the dysphoric and anxiosomatic circuits^12^, and tested for between-group differences using unpaired t-test or non-parametric Mann-Whitney U test, in the presence of outliers.

**Figure 1:**
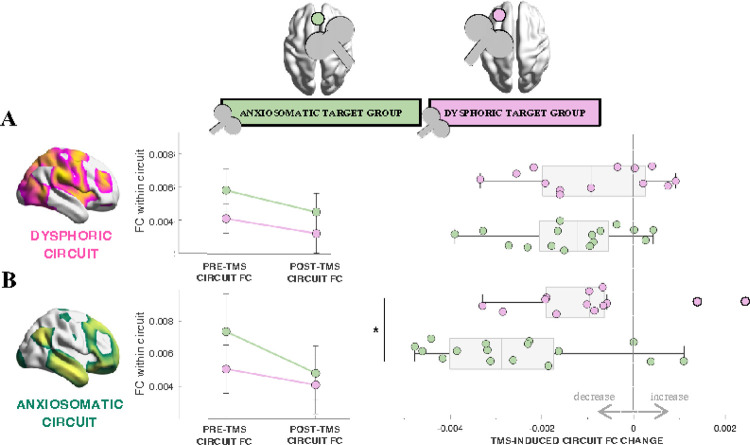
TMS at the anxiosomatic target induced greater FC decrease within the anxiosomatic circuit. **(A, B *left*):** pre- and post-TMS group mean and standard deviation FC values within the dysphoric (*A*) and anxiosomatic (*B*) circuits are plotted across target groups. **(A, B *right*):** the TMS-induced FC change within dysphoric (*A*) and anxiosomatic (*B*) circuits is depicted as boxplots across target groups. Significant group differences (p<0.05) in FC change are indicated by an asterisk. FC: functional connectivity; TMS: transcranial magnetic stimulation.

Results partially confirmed our *a priori* hypothesis. While we found no significant between-group difference in FC decrease within the dysphoric circuit (unpaired t-test, *t*=0.85, *p*=0.40, Figure 1A), FC within the anxiosomatic circuit decreased more in the anxiosomatic target group compared to the dysphoric target group (Mann-Whitney U test, *z*=2.30, *p*=0.02, Figure 1B). However, post hoc analyses revealed a significant baseline difference between groups that could be driving the effect, with patients randomized to the anxiosomatic target displaying higher baseline FC within both dysphoric (*z*=−3.35, *p*=0.0004) and anxiosomatic (*z*=−2.78, *p*=0.005) circuits. When controlling for baseline FC in a linear regression model, we found no effect of target group on FC change within the anxiosomatic circuit (*F(1,26)*=0.38, *p*=0.54). Results were unchanged when using an alternative method to computing circuit-based FC (Supplementary Results).

In summary, while TMS at the dysphoric target did not induce greater FC decrease within the dysphoric circuit, TMS at the anxiosomatic target induced greater FC decrease within the anxiosomatic circuit. However, this effect seems to be driven by baseline FC: the higher the baseline FC within the anxiosomatic circuit, the greater the TMS-induced FC decrease (*F(1,26)*=13.28, *p*=0.001).

### TMS site FC to the targeted circuit correlates with FC changes

In the head-to-head trial, patients received TMS at group-level target coordinates previously linked to improvement in either anxiosomatic or dysphoric symptoms.^7^ While derived from population data, the degree to which each patient’s TMS site is functionally connected to an underlying circuit varies incidentally, creating a natural experiment. We hypothesized that stronger baseline FC between an individual’s stimulation site and a circuit would correlate with greater FC changes within that circuit. The *a priori* linear regressionmodel assessing this effect included baseline FC to the opposite circuit and target group as covariates to isolate target-specific effects.

**Figure 2:**
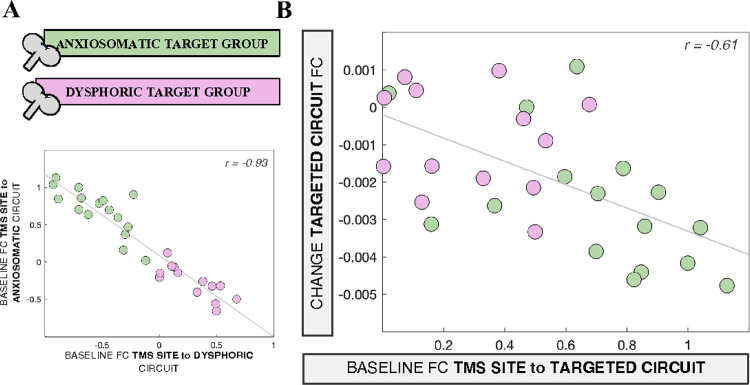
TMS site FC to the targeted circuit predicts FC change within that circuit. **(A):** Baseline TMS site FC to the dysphoric circuit is plotted against baseline TMS site FC to the anxiosomatic circuit across the two target groups, showing strong collinearity between these variables. **(B):** Baseline TMS site FC to the circuit specifically targeted in each group (i.e., dysphoric circuit for the dysphoric target group, and vice versa) is plotted against TMS-induced FC change of the targeted circuit. FC: functional connectivity; TMS: transcranial magnetic stimulation.

In the *a priori* analysis, baseline FC to the anxiosomatic circuit did not predict change within the anxiosomatic circuit (*F(1,25)*=1.99, *p*=0.17, *partial R*=0.26), nor did FC to the dysphoric circuit predict change within the dysphoric circuit (*F(1,25)*=0.01, *p*=0.91, *partial R*=0.1). However, post-hoc analyses revealed high collinearity between baseline FC to the two circuits (*r*=−0.93, *p*=1.6×10^−7^, VIF_DYS_=8.69; VIF_ANX_=8.23) (Figure 2A), suggesting that our *a priori* decision to include both as independent predictors in the model was flawed.

We therefore performed a post-hoc analysis considering the circuit respectively targeted in each group. We found a significant correlation between baseline FC to the targeted circuit and FC change within that circuit (*r*=−0.61, *p*=6.2×10^−4^, Figure 2B). This effect was primarily driven by the anxiosomatic group (*r*=−0.62, p=0.01), and was not driven by variability in baseline FC, as the result remained significant when controlling for baseline FC within the anxiosomatic circuit (*r*=−0.59, *p*=0.02). No significant relationship was observed in the dysphoric group alone, although the trend was in a similar direction (*r*=−0.22, *p*=0.47; *r*=0.20, *p*=0.53 after controlling for baseline FC within the dysphoric circuit). Notably, these results are consistent with the preliminary findings that informed our hypotheses.

We also repeated the analyses with different approaches to modeling the TMS site, as a 5mm sphere or as decaying electric fields. We again found a significant correlation between baseline FC to the targeted circuit and FC change within that circuit, driven by the anxiosomatic group (**Table S1**).

In summary, stronger baseline FC between the TMS site and the targeted circuit was associated with greater FC decrease within that circuit. This effect was not independent of the effect of TMS site FC to the non-targeted circuit, as there was significant collinearity between the two circuits. The result was driven primarily by the anxiosomatic group.

### Baseline TMS site FC, not FC change, correlates with symptom improvement

Lastly, we hypothesized that baseline TMS site FC to a circuit, and FC change within a circuit, would correlate with symptom improvement, measured as changes in Beck Depression Inventory (BDI) and Beck Anxiety Inventory (BAI) scores. Correlations were calculated separately for each target group and pooled into a combined coefficient, weighted by the respective sample sizes. Results across different TMS site models, and correlation values for each group, are reported in **Table S2** and **S3**.

**Figure 3:**
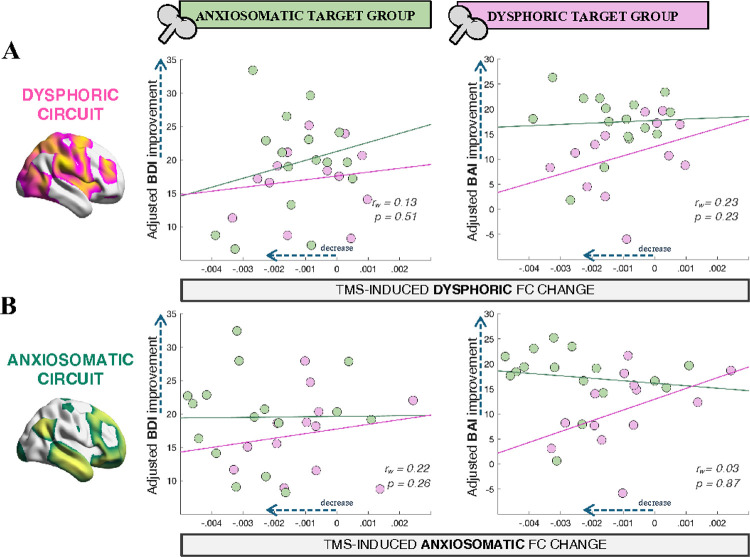
TMS-induced decrease in circuit FC does not predict clinical outcome. **(A):** TMS-induced FC change within the dysphoric circuit is plotted against BDI (*left*) and BAI (*right*) difference from baseline, after regressing the effects of age, sex, baseline symptoms, baseline circuit FC and improvement on the opposite symptom scale. **(B):** TMS-induced FC change within the anxiosomatic circuit is plotted against BDI (*left*) and BAI (*right*) difference from baseline, after adjusting for covariates. BDI: Beck Depression Inventory; BAI: Beck Anxiety Inventory; TMS: transcranial magnetic stimulation; r_w_: Spearman correlation coefficient across groups, weighted by their respective sample size.

We found no significant association between FC changes and symptom improvement: FC changes within the dysphoric circuit did not correlate with change in BDI (*r*_*w*_=0.13, *p*=0.51) or BAI (*r*_*w*_=0.23, *p*=0.23) (Figure 3A), and FC changes within the anxiosomatic circuit did not correlate with BDI (*r*_*w*_=0.22, *p*=0.26) or BAI (*r*_*w*_=0.03, *p*=0.87) improvement (Figure 3B).

**Figure 4:**
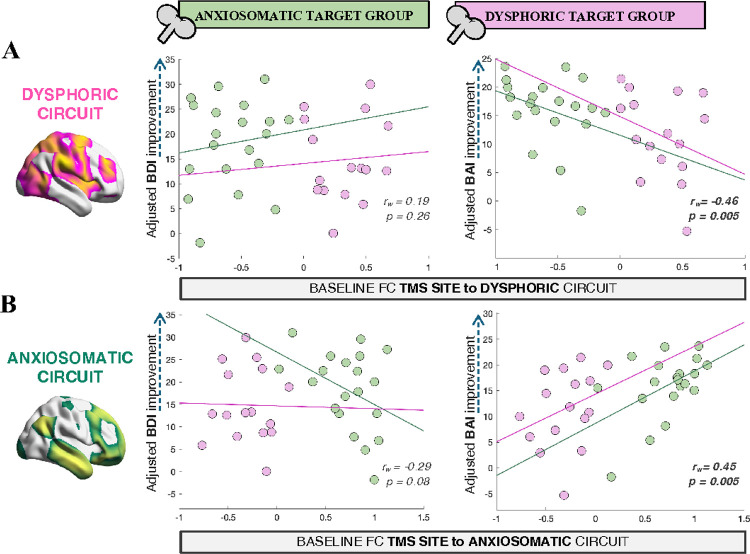
Baseline TMS site FC to circuit predicts anxiety improvement. **(A):** Baseline TMS site FC to the dysphoric circuit is plotted against BDI (*left*) and BAI (*right*) difference from baseline, after regressing the effects of age, sex, baseline symptoms, and improvement on the opposite symptom scale. **(B):** Baseline TMS site FC to the anxiosomatic circuit is plotted against BDI (*left*) and BAI (*right*) difference from baseline, after adjusting for covariates. Significant correlations are indicated in bold. BDI: Beck Depression Inventory; BAI: Beck Anxiety Inventory; TMS: transcranial magnetic stimulation; r_w_: Spearman correlation coefficient across groups, weighted by their respective sample size.

In contrast, greater BAI improvement was associated with stronger TMS site FC to the anxiosomatic circuit (*r*_*w*_=0.45, *p*=0.005), and with weaker FC to the dysphoric circuit (*r*_*w*_=−0.46, *p*=0.005), controlling for age, sex, baseline symptoms and change in the opposite symptom scale(Figure 4). No significant correlation was found between BDI improvement and FC to the dysphoric (*r*_*w*_=0.19, *p*=0.26) or anxiosomatic circuit (*r*_*w*_=−0.29, *p*=0.08), although the trends were in the same direction of the anxiosomatic circuit results. These analyses on baseline FC included the 36 patients who completed the intention-to-treat endpoint.

In summary, TMS-induced FC changes within either circuit were not associated with clinical outcomes. Instead, baseline FC of the stimulation site to either circuit correlated significantly with anxiety improvement in the expected direction – stronger FC to the anxiosomatic circuit and weaker FC to the dysphoric circuit were associated with greater anxiety relief.

### Post-hoc analyses on circuit properties

**Figure 5:**
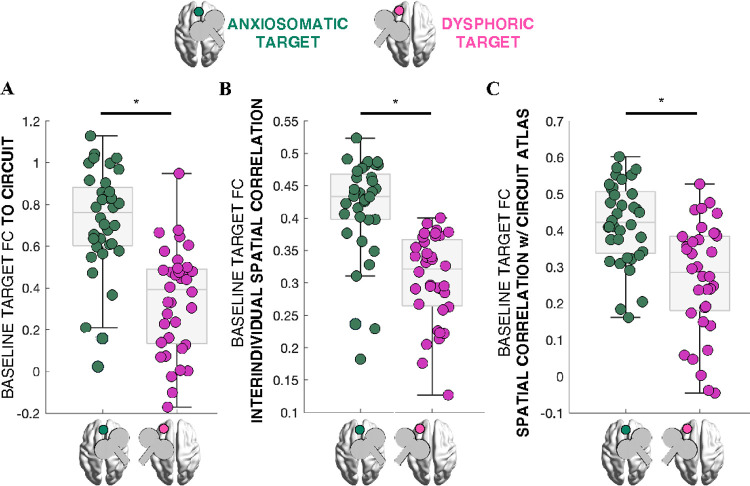
The anxiosomatic and dysphoric circuit targets have different properties. **(A):** Baseline functional connectivity (FC) between the anxiosomatic target and the anxiosomatic circuit (green) compared to FC between the dysphoric target and the dysphoric circuit (pink). **(B):** Mean spatial correlation of each individual’s whole-brain FC map (seeded from either the anxiosomatic or dysphoric target) with the corresponding maps from all other participants. **(C):** Spatial correlation between each individual’s whole-brain FC map (from the anxiosomatic or dysphoric target) and the corresponding group-level circuit atlas. Significant group differences are indicated with an asterisk (*).

Given that significant effects were observed specifically for the anxiosomatic circuit, we conducted post-hoc analyses to investigate potential differences in circuit properties. We assessed whether the anxiosomatic circuit was targeted more consistently: across the full sample (N=36), baseline FC between the anxiosomatic target site and the anxiosomatic circuit was significantly stronger than FC between the dysphoric target site and the dysphoric circuit (Wilcoxon Signed Rank Test *z*=4.69, *p*=2.6×10^−6^, Figure 5A).

This finding raised the possibility that the group-based anxiosomatic target may be more representative of individual connectivity profiles, potentially due to lower inter-subject variability in circuit FC. To test this, we computed seed-based FC from the group-based targets and found that whole-brain FC of the anxiosomatic target was more similar across individuals than that of the dysphoric target (z=4.95, p=7.5×10^−7^, Figure 5B). Additionally, individual whole-brain FC maps of the anxiosomatic target showed greater similarity to the group-level atlas of the anxiosomatic circuit than the dysphoric target FC maps did to the dysphoric circuit (z=4.27, p=1.9×10^−5^, Figure 5C).

## Discussion

In this pre-specified clinical trial, we assessed whether directly targeting a dysphoric or an anxiosomatic circuit would selectively reduce FC within the targeted circuit, and whether this would be related to baseline TMS site FC to that circuit, and to clinical response to TMS.

Our findings partially supported our hypotheses. We observed target-dependent decreases in FC within the anxiosomatic circuit, although this effect was confounded by group differences in baseline FC. The magnitude of FC change was associated with the strength of baseline FC between the TMS site and the target circuit. Importantly, this result was consistent with preliminary analyses in an independent cohort of patients. However, contrary to our expectations, FC changes did not correlate with symptom improvement. Rather than with changes in FC following treatment, greater anxiety relief was associated with stronger baseline FC between the TMS site and the anxiosomatic circuit.

These findings have important translational implications. In clinical trials, target engagement is typically measured to investigate the relationship between clinical improvement and a hypothesized mechanism of action. While numerous studies have linked TMS to various brain signal changes,^6^ the clinical utility of such biomarkers may depend on their specificity to a given target and their ability to predict change in symptoms. Even when FC changes are shown to be target-dependent and/or predictive of symptom improvement, implementing this approach as a biomarker of effective treatment presents practical challenges – it would require repeated FC measurements throughout treatment and potentially interrupting or altering the treatment course if no change is observed. In contrast, baseline FC between the TMS site and a target circuit can be measured prior to initiating treatment, offering a more actionable strategy.

These results align within a broader body of research leveraging FC to optimize TMS targeting. Work by Fox et al. (2012) first suggested that clinical response to TMS is associated with FC between the left dlPFC and the subgenual cingulate.^1^ This relationship has since been replicated across multiple studies,^9,13–15^ and it is sometimes considered one of the most robust rs-fMRI-based biomarkers for predicting TMS response.^5^ As the field has shifted from focusing on isolated brain regions to broader, circuit-based models,^16^ studies have shown that connectivity between stimulation sites and distributed circuits can better predict clinical response to TMS.^17,18^ Our findings further support this notion.

Notably, baseline FC between the anxiosomatic target and the anxiosomatic circuit was significantly stronger than that between the dysphoric target and its circuit. This suggests that, even with group-level targeting, the anxiosomatic circuit can be more consistently targeted. One possible explanation for this difference lies in the intrinsic properties of the two circuits. Both were derived from a data-driven clustering analysis of symptom-specific TMS outcomes,^7^ but the dysphoric circuit was defined by clustering across more symptoms, potentially introducing more heterogeneity and noise into the resulting circuit map. Spatially, the dysphoric circuit spans multiple large-scale networks, including the ventral and dorsal attention networks, and frontoparietal control network, and recent evidence suggests that it might be further subclassified.^19^ In contrast, the anxiosomatic circuit was based on fewer symptoms and largely overlaps with the default mode network, potentially resulting in a more spatially coherent and homogeneous circuit architecture across individuals. Notably, prior evidence indicates lower interindividual variability in functional network topography within the medial versus lateral prefrontal cortices.^20^

Post-hoc analyses support these interpretations. Whole-brain FC seeded from the group-based anxiosomatic target was more similar across individuals than dysphoric target FC, and it showed greater similarity to the group-level circuit atlas. As a result, despite using group-level rather than individualized targeting, stimulation at the anxiosomatic target may have more closely approximated individual optimal stimulation sites, and led to greater engagement of the anxiosomatic circuit. The clinical trial’s primary and secondary outcomes demonstrated that, while both targets were equally effective in reducing depressive symptoms overall, the anxiosomatic target led to greater improvement in anxiety symptoms. These observed clinical benefits might be driven by more consistent targeting and engagement of the anxiosomatic circuit, and highlight the importance of considering circuit architecture and variability when choosing between a group-based vs individualized approach.

Key strengths of this study include its pre-specified approach within a randomized trial, and the use of individualized rs-fMRI data. However, a few limitations need to be considered. First, this study included a relatively small sample. Although it was adequately powered to detect the hypothesized effects, and it was built upon results from an independent cohort, validation in larger, independent datasets is needed. Second, in some instances our *a priori* approach was misspecified, and some of our conclusions rely on post-hoc analyses conducted to better interpret the outcomes of the pre-specified analyses. Namely, our pre-specified analysis showed a greater FC decrease within the anxiosomatic circuit in the anxiosomatic group, but post-hoc analyses suggested this result was confounded by baseline differences. Next, the assumption of independent effects of TMS site FC to each circuit proved invalid due to strong collinearity. A post-hoc analysis addressing this, however, revealed a robust association between TMS site FC to the targeted circuit and FC change within that circuit.

## Conclusions

Individual variability in FC between the stimulation site and an underlying brain circuit was associated with both functional engagement of that circuit and with symptom improvement. The anxiosomatic circuit was more consistently targeted and better engaged than the dysphoric circuit, likely due to lower interindividual variability in its architecture. However, FC changes did not predict symptom improvement. Instead, stronger baseline FC between the TMS site and the targeted circuit was associated with greater anxiety relief, suggesting it may serve as a more reliable and actionable biomarker for informing TMS treatment. These findings highlight the importance of accounting for circuit architecture and interindividual variability when developing connectivity-guided TMS protocols. Future studies should aim to replicate these findings in larger samples, and evaluate the advantage of individualized targeting.

## Methods

### Sample characteristics and study procedures

Details about the inclusion criteria, study procedure, primary and secondary outcomes are described elsewhere^8^. Briefly, this phase-2, double-blind, parallel-group randomized trial recruited adults (18–65yo) meeting FDA criteria for TMS for depression, with a Beck Depression Inventory (BDI)^21^ score of 20 or higher, and a Beck Anxiety Inventory (BAI)^22^ score of 16 or higher. Before treatment, participants completed behavioral tasks, questionnaires, structural (TR=2.3s, 1×1×1mm) and functional (four 7.5-min runs, TR=0.8s, 2.4×2.4×2.4mm) MRI on a 3T Siemens Prisma scanner. Participants were then randomized to either the dysphoric (MNI: [−32,44,34]) or anxiosomatic (MNI: [0,48,46]) TMS target. All participants received TMS following the 2008 FDA cleared protocol (3000 pulses per treatment, 4 s trains, 26 s inter-train interval, 10 Hz frequency, 120% resting motor threshold), using neuronavigation to the group-based target and a robotic arm for coil placement. At the end of treatment, a post-TMS visit identical to the baseline visit took place. The primary clinical outcome of the trial was symptom-specific changes (as measured by the ratio of change in depression to anxiety). Secondary outcomes included group differences in TMS effects on depression and anxiety alone. Out of the 40 patients randomized to either TMS target, N=36 patients completed the intention-to-treat endpoint (i.e., at least 20 sessions), and N=29 patients completed the post-TMS imaging visit.

### Image preprocessing and functional connectivity measures

Individual structural and functional scans were preprocessed following previously established methods^23–26^. Briefly, resting-state fMRI preprocessing steps included: removal of the first four volumes of each run, head motion outliers correction, registration across runs, linear regression of motion, whole brain, white matter, ventricle and a first-order polynomial trend, motion censoring (for a Framewise Displacement greater than 0.2mm and DVARS greater than 50), bandpass filtering (0.001 to 0.08Hz), spatial smoothing with 6mm full width half maximum kernel, normalization to the MNI 2mm template and concatenation of different runs. Preprocessed data were imported into the CONN toolbox (version 22a)^27^ to compute resting-state FC measures.

We modelled the TMS site as a 12mm decaying sphere centered at MNI coordinates of the dysphoric and anxiosomatic targets, as in our prior work.^28^ For validation, we repeated our analyses using alternative models of the TMS site, defined as a 5mm sphere and as the TMS-induced electric-field, simulated using SimNIBS (v4.0.1, https://simnibs.github.io) and thresholded to include only the top 0.1%, 1% or 5% of strongest voxels. We employed the publicly available statistical maps of the dysphoric and anxiosomatic circuits derived in the original study (https://identifiers.org/neurovault.image:787858).^7^ The TMS site was excluded from the circuit maps to avoid circularity in subsequent analyses.

To assess target engagement, we computed the change in average FC within the anxiosomatic and dysphoric circuits, as the weighted mean of all pairwise correlation coefficients between voxels within each circuit. Weights were derived from the voxel-wise statistical values in the original circuit masks^12^.

We computed baseline FC between the TMS site and anxiosomatic and dysphoric circuits as the weighted Fisher-transformed bivariate correlation coefficient between the average timecourse of each region of interest. Weights were derived from the voxel-wise statistical values of the circuit masks, and the voxel-wise intensity of the TMS model representing the relative strength of stimulation.

In post-hoc analyses, we additionally computed seed-based FC maps by calculating the Fisher-transformed bivariate correlation between the average BOLD timeseries of the TMS site (modeled as a 12mm decaying sphere at the anxiosomatic or dysphoric target) and the timeseries of every other voxel in the brain.

### Statistical Analyses

All statistical analyses were performed using Matlab (R2022a, MathWorks Inc, Natick, MA). Preliminary analyses on an independent cohort of patients (N=18) undergoing clinical TMS informed the hypotheses and statistical approach for the current study.

#### Target-dependent changes in FC within circuits.

First, we hypothesized that, compared to TMS to the dysphoric target, TMS to the anxiosomatic target would induce greater reduction in FC within the anxiosomatic circuit, and vice versa. Between-group differences in FC change within each circuit were assessed using two-tailed unpaired t-tests. In the presence of outliers or violations of normality, we employed the Mann-Whitney U test, a non-parametric method more robust to non-normal data distributions. Post-hoc analyses assessed whether baseline FC within each circuit differed by group, and whether baseline circuit FC would influence the magnitude of FC change. This was tested using linear regression models, with FC change within each circuit as dependent variable, and baseline circuit FC and target group as predictors.

#### Effect of baseline TMS site FC on circuit FC change.

Second, we hypothesized that baseline FC between the TMS site and a given circuit would predict the magnitude of FC change within that circuit, independently of TMS site FC to the opposite circuit. In line with our pre-specified approach, we tested this effect using linear regression models with FC change within each circuit as the dependent variable, and baseline TMS site FC to that circuit as predictor of interest. Baseline TMS site FC to the opposite circuit and target group were added as control predictors to the model. To mitigate the influence of outliers, all predictors and outcomes were rank-transformed. In post-hoc analyses, we assessed collinearity between predictors, by computing their correlation and variance inflation factors (VIF). To address strong collinearity, we then computed the Spearman’s correlation between baseline TMS site FC to the targeted circuit (i.e., anxiosomatic circuit for the anxiosomatic target group, dysphoric circuit for the dysphoric target group) and FC change within that circuit. Thus, in contrast to the pre-specified model, this post-hoc analysis omitted the non-targeted circuit and group identity as covariates, as the analysis was restricted to the circuit targeted in each group. We however additionally computed correlation coefficients in each group separately, and additionally controlled for baseline circuit FC.

#### Relationship with symptom improvement.

Lastly, we hypothesized that baseline TMS site FC to a circuit and TMS-induced FC changes within a circuit would correlate with treatment outcomes. Treatment outcome was defined as change from baseline to the end of treatment in depression (BDI) and anxiety (BAI) scores. Associations were tested using Spearman’s partial correlations, including age, sex, baseline symptom severity, and change scores on the opposite symptom scale as covariates. Models testing the association between circuit FC change and clinical outcome also included baseline circuit FC as a covariate. To assess the overall association across target groups while accounting for their respective sample sizes, correlations were calculated separately within each group, and subsequently aggregated into a sample-size weighted average of each Fisher-transformed correlation coefficient. Models testing the association with baseline TMS site FC included the 36 patients who completed the intention-to-treat endpoint. All hypothesis tests were two-tailed.

#### Post-hoc analyses on circuit properties.

To assess systematic differences between the two targets, we compared baseline FC between the anxiosomatic target and the anxiosomatic circuit to baseline FC between the dysphoric target and the dysphoric circuit. To evaluate differences in inter-individual variability in circuit connectivity, we computed the mean spatial correlation between each individual’s whole-brain FC map – seeded from either the anxiosomatic or dysphoric target – and the corresponding maps from all other participants. To quantify alignment with the circuit template, we also computed the spatial correlation between each individual’s whole-brain FC map of the anxiosomatic and dysphoric target with the corresponding group-level circuit atlas (https://identifiers.org/neurovault.image:787858). All analyses included data from the 36 patients who completed the intention-to-treat endpoint, regardless of target group assignment. Wilcoxon Signed Rank Tests were used to test for between-circuit differences while accounting for potential outliers.

## Supplementary Material

Supplementary Files

This is a list of supplementary files associated with this preprint. Click to download.

- SupplementaryMaterial.docx
